# Neuronal loss and microgliosis are restricted to the core of Aβ deposits in mouse models of Alzheimer's disease

**DOI:** 10.1111/acel.13380

**Published:** 2021-05-25

**Authors:** Jing Zhang, Na Wu, Shubo Wang, Zitong Yao, Fuchuan Xiao, Jing Lu, Baian Chen

**Affiliations:** ^1^ School of Basic Medical Sciences Beijing Key Laboratory of Neural Regeneration and Repair Capital Medical University Beijing China; ^2^ Laboratory Animal Resource Center Capital Medical University Beijing China

**Keywords:** Alzheimer's disease, axon loss, Aβ deposits, microglia, myelin sheath loss, neuron loss

## Abstract

Amyloid‐β (Aβ) deposits, pathologic tau, and neurodegeneration are major pathological hallmarks of Alzheimer's disease (AD). The relationship between neuronal loss and Aβ deposits is one of the fundamental questions in the pathogenesis of AD. However, this relationship is controversial. One main reason for the conflicting results may be the confounding effects of pathologic tau, which often coexists with Aβ deposits in the brains of AD patients. To clarify the relationship between neuronal loss and Aβ deposits, mouse models of AD, which develop abundant Aβ deposits in the aged brain without pathologic tau, were used to examine the co‐localization of NeuN‐positive neurons, NF‐H‐positive axons, MBP‐positive myelin sheaths, and Aβ deposits. Neuronal loss, as measured by decreased staining of the neuronal cell body, axon, and myelin sheath, as well as the IBA‐1‐positive microglia, was significantly increased in the core area of cerebral Aβ deposits, but not in adjacent areas. Furthermore, neuronal loss in the core area of cerebral Aβ deposits was correlated with Aβ deposit size. These results clearly indicate that neuronal loss is restricted to the core of Aβ deposits, and this restricted loss probably occurs because the Aβ deposit attracts microglia, which cluster in the core area where Aβ toxicity and neuroinflammation toxicity are restrained. These findings may contribute to our understanding of the relationship between neuronal loss and Aβ deposits in the absence of pathologic tau.

## Graphical abstract

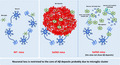

## 1 INTRODUCTION

Alzheimer's disease (AD) is the most common type of dementia and the risk of AD increases greatly with advancing age. Cerebral amyloid‐β (Aβ) deposits, pathologic tau, and neurodegeneration are major pathological hallmarks of AD (Jack et al., 2018). One of the fundamental questions concerning the etiology of AD is whether Aβ deposits are a causative agent leading to neuronal loss or coincide with neuronal loss caused by a common agent (Vandenberghe, 2014). The idea that Aβ deposits are upstream pathological factors that lead to pathologic tau and the neuronal loss responsible for cognitive impairment is supported by many publications (Hardy & Selkoe, 2002; Hayden & Teplow, 2013; Selkoe & Hardy, 2016). However, other publications do not support the central role of Aβ deposits in AD (Eimer et al., 2018; Makin, 2018; Morris et al., 2018). Some of the controversy regarding the role of Aβ deposits in AD originates from the presence of Aβ deposits in normal adults (Eimer et al., 2018; Makin, 2018; Morris et al., 2018; Rodrigue et al., 2009). While Aβ deposits are present in AD patients, they are also present in approximately 30% of adults with no cognitive impairment (Dickson et al., 1992). The relationship between Aβ deposits and neuronal loss remains controversial. While several studies reported that Aβ deposits are not well correlated with neuronal loss or brain atrophy, other studies yielded conflicting results (Josephs et al., 2008; La Joie et al., 2020; Pulina et al., 2020). Many factors may contribute to this discrepancy. One confounding factor is the coincidental presence of pathologic tau, which is associated with induction of neuronal loss. Aβ deposits and pathologic tau coexist in the brains of most AD patients (Fu et al., 2017; Jansen et al., 2015; La Joie et al., 2020). Therefore, pathologic tau influences the results of studies of the relationship between Aβ deposits and neuronal loss or brain atrophy. Thus, while pathologic tau has been established as a cause of neuronal dysfunction in AD, the effects of Aβ deposits on neuronal dysfunction are less certain.

Researchers have generated several mouse models carrying human amyloid protein precursor (APP) and/or presenilin (PSEN) transgenes with different AD‐linked mutations, including 5xFAD, APPswe/PSEN1dE9 (Garcia‐Alloza et al., 2006), APPswe/PSEN1(A246E) (Borchelt et al., 1997), Tg 2576 (Hsiao et al., 1996), etc., which develop Aβ deposits lacking pathogenic tau in the brain. These mouse models provide good opportunities to study the relationship between Aβ deposits and neuronal loss without the confounding effects of pathogenic tau (Borchelt et al., 1997; Garcia‐Alloza et al., 2006; Hsiao et al., 1996; Jawhar et al., 2012; Oakley et al., 2006). Some of these mice, including 5xFAD (Jawhar et al., 2012; Oakley et al., 2006) and APPswe/PSEN1dE9 (Ma et al., 2017) mice, shown significant neuron loss in the brain, whereas others, such as APPswe/PSEN1(A246E) (Borchelt et al., 1997) and Tg 2576 (Hsiao et al., 1996) mice, show either absent or very limited neuron loss in the brain. These studies suggest that the effects of Aβ deposits on neuron loss in the brain are complicated and remain unclear. Among these mouse models, the Aβ deposits develop in the brains of 5xFAD mice after as little as around 3–6 months, and these accumulations eventually develop into abundant Aβ deposits, with no pathologic tau, in the aged brain (Jawhar et al., 2012; Oakley et al., 2006). Thus, the 5xFAD mouse is an ideal model for studies of the relationship between Aβ deposits and neuronal loss because of its well‐defined Aβ pathology and because it is insusceptible to the confounding effects of pathologic tau. Jawhar (Jawhar et al., 2012) and others (Oakley et al., 2006) reported that there was neuron loss in the cortical layer V and subiculum, but not in other brain regions of 5xFAD mice, but they did not confirm the relationship between Aβ deposits and neuron loss. Later, Eimer and Vassar reported (Eimer & Vassar, 2013) that the loss of NeuN‐positive neurons correlates well with intraneuronal Aβ42 accumulation in the 5xFAD mouse, but they did not analyze the relationship between extracellular Aβ deposits and neuron loss. In addition, previous studies (Eimer & Vassar, 2013; Jawhar et al., 2012; Oakley et al., 2006) did not analyze the neuron number in the core area of Aβ deposits, which probably explains why neurons were reported to be lost in some brain regions, like cortical layer V and the subiculum, but not in other regions. Aβ readily accumulates together to form deposits and is not widely homogeneously distributed, so it is likely that the toxicity of Aβ on neurons is limited on the core areas of deposits in the brain rather than peripheral areas. In brief, the relationship between Aβ deposits and neuronal loss has not been fully elucidated yet.

The aim of this study was to determine the association between Aβ deposits and markers of neuronal loss in the 5xFAD mouse. We determined the co‐localization of Aβ deposits with markers of neuron cell bodies, neurofilament alterations, and myelin sheath degradation in mouse brain tissue slices, as well as with marker of microgliosis. We also demonstrated the correlation of Aβ deposit size with neuronal loss. Our data clearly show that neuronal loss (including neuronal, axonal, and myelin sheath loss) occurred in the core areas of Aβ deposits and not in the surrounding tissue, which was probably due to the attraction of increased microglia clusters by Aβ deposits in the core area, where Aβ toxicity and neuroinflammation toxicity were restrained. Furthermore, neuronal loss was correlated with the size of Aβ deposits.

## 2 RESULTS

### 2.1 NeuN‐positive neuron loss is restricted to the core area of Aβ deposits

**FIGURE 1:**
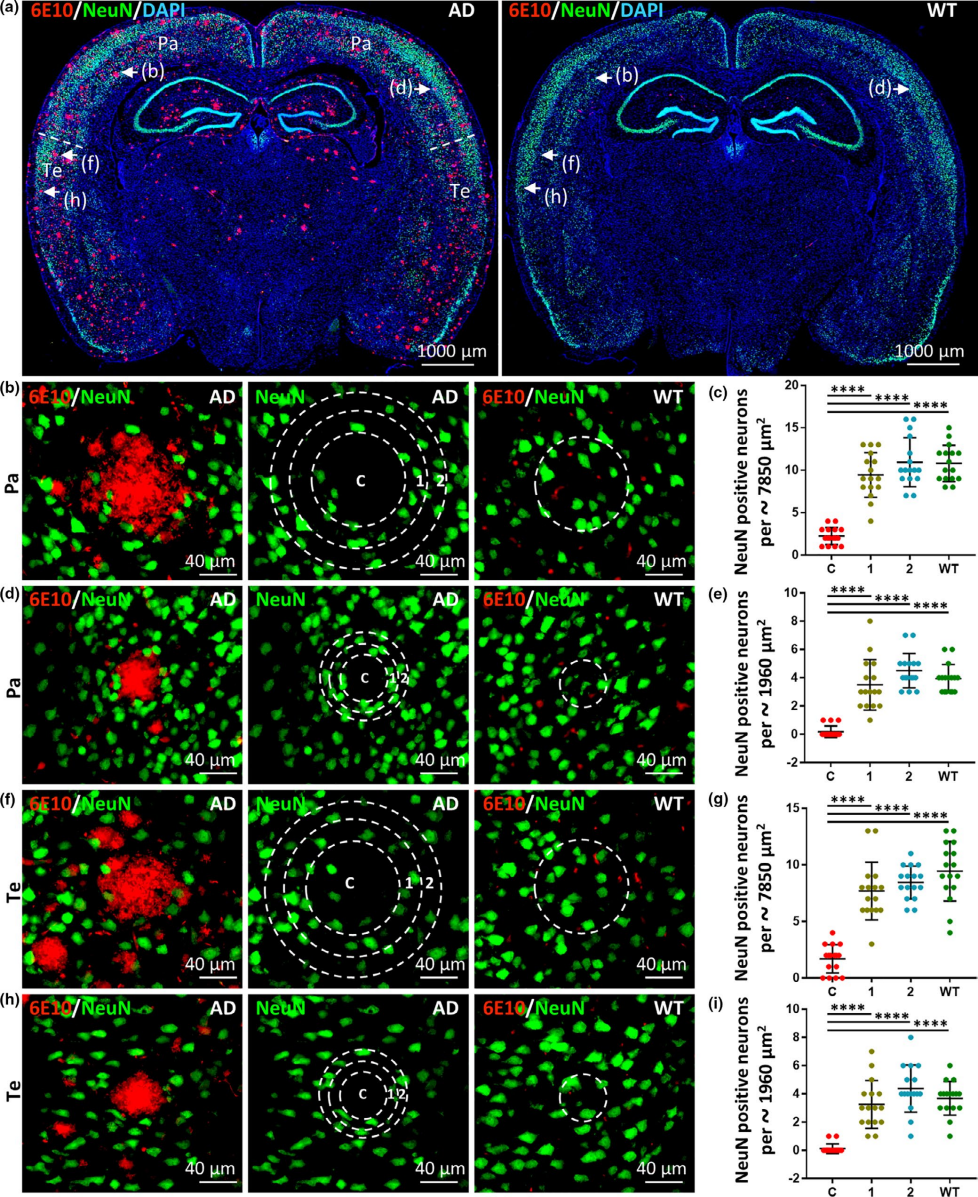
NeuN‐positive neuronal loss is restricted to the core area of Aβ deposits. (a) Representative images of double‐label immunofluorescence staining in coronal brain sections from 12‐month‐old 5xFAD and WT mice. Aβ deposits, labeled with anti‐Aβ antibody (6E10) and Alexa Fluor 647 goat anti‐mouse secondary antibody, are shown in red. NeuN‐positive neurons, labeled with anti‐NeuN antibody and Alexa Fluor 488 goat anti‐rabbit secondary antibody, are shown in green. Nuclei were counterstained with DAPI and are shown in blue. Scale bars, 1000 µm. Representative images of Aβ deposits in the parietal (Pa) and temporal cortex (Te) from 5xFAD and WT mice were taken with high magnification (b, d, f, and h). Aβ deposits of approximately 100 µm (b and f) and 50 µm (d and h) in diameter in the Pa or Te, respectively, in 5xFAD mice are shown and outlined by a core circle (C) with two adjacent concentric rings (1, 2) of the same area. Circular regions corresponding to the same areas are shown in WT mice. Scale bars, 40 µm. The NeuN‐positive neurons in the C, 1, and 2 regions were counted and compared (c, e, g, and i). Quantitative analyses of the number of NeuN‐positive neurons present in the core circle (C) and two adjacent concentric rings (1, 2) of 5xFAD mice and the corresponding circular region of WT mice (labeled as WT) are shown. Compared with NeuN‐positive neurons in the two adjacent concentric rings (1, 2) and WT mice, the number of NeuN‐positive neurons present in the core circle decreased drastically. Data derived from sixteen images of eight different mice for each group (*n* = 16). Data represent mean ±SD. ****p < 0.0001

Previous clinical imaging studies and postmortem studies of AD patients were focused on the relationship between neuronal loss and Aβ deposits, but were inevitably affected by the confounding presence of pathologic tau. We used the 5xFAD mouse to clarify the relationship between neuronal loss and Aβ deposits without the influence of pathologic tau. 5xFAD mice carry AD pathogenic gene mutations that lead to the development of abundant Aβ deposits of different sizes in the brain (Figure 1a), but they do not express pathologic tau (Figure S1). To investigate the effects of endogenous Aβ deposits on the number of neurons in the brain, we compared the number of neurons in the areas of Aβ deposits to adjacent areas with no Aβ deposits in 5xFAD and wild type (WT) mice at the age of 12 months. Monoclonal anti‐Aβ antibodies (6E10) that recognize Aβ deposits (red) were used in conjunction with anti‐NeuN antibodies to label neuron cell bodies (green) (Figure 1). Significantly fewer NeuN‐positive neurons were found in the core area of Aβ deposits in the parietal (Pa) and temporal cortex (Te) in comparison with the two continuous adjacent areas in 5xFAD mice and the corresponding areas of age‐matched WT mice (Figure 1b and c, C vs. 1, *p* < 0.0001; C vs. 2, *p* < 0.0001; C vs. WT, *p* < 0.0001. Figure 1d and e, C vs. 1, *p* < 0.0001; C vs. 2, *p* < 0.0001; C vs. WT, *p* < 0.0001. Figure 1f and g, C vs. 1, *p* < 0.0001; C vs. 2, *p* < 0.0001; C vs. WT, *p* < 0.0001. Figure 1h and i, C vs. 1, *p* < 0.0001; C vs. 2, *p* < 0.0001; C vs. WT, *p* < 0.0001). The number of NeuN‐positive neurons in areas adjacent to Aβ deposits in 5xFAD mice was not significantly different from that in the corresponding areas in age‐matched WT mice (Figure 1b‐i, 1 vs. WT, *p* > 0.05; 2 vs. WT, *p* > 0.05; 1 vs. 2, *p* > 0.05).

Neuron loss in the core area of Aβ deposits may be due to Aβ toxicity or growth of deposits to replace the space previously used by neurons. Since the release of cytochrome C (Cyt C) is a common feature during cellular death and MAP2 loss is a classic marker for neurodegeneration, we measured the number of abnormal Cyt C‐positive aggregates and the intensity of MAP2 in the core area and two adjacent areas of Aβ deposits in the Pa of 12‐month‐old 5xFAD mice and the corresponding area of age‐matched WT mice to verify whether neurons died or were replaced in the core area of Aβ deposits (Figure S2). The results show that the number of abnormal Cyt C‐positive aggregates in the core area of Aβ deposits in the Pa was significantly increased in comparison with that of the two adjacent areas in 5xFAD mice and in the corresponding area of age‐matched WT mice (Figure S2a, b, and d, C vs. 1, *p* < 0.0001; C vs. 2, *p* < 0.0001; C vs. WT, *p* < 0.0001). The intensity of MAP2‐positive dendrites in the core area of Aβ deposits in the Pa was significantly decreased in comparison with that of the two adjacent areas in 5xFAD mice and the corresponding area of age‐matched WT mice (Figure S2a, c, and e, C vs. 1, *p* < 0.001; C vs. 2, *p* < 0.0001; C vs. WT, *p* < 0.0001). There were no significant differences in the number of abnormal Cyt C‐positive aggregates or the intensity of MAP2‐positive dendrites between the areas adjacent to the Aβ deposits in 5xFAD mice and the corresponding area of age‐matched WT mice (Figure S2, 1 vs. WT, *p* > 0.05; 2 vs. WT, *p* > 0.05; 1 vs. 2, *p* > 0.05). These results indicate that cellular death and neurodegeneration occurred in the core area of Aβ deposits. In addition, we analyzed the neuron numbers in the cortex of young 5xFAD mice (5 and 8 months) and age‐matched WT mice (Figure S3). As observed in 12‐month‐old 5xFAD mice (Figure 1), NeuN‐positive neuron loss was restricted to the core area of Aβ deposits in both 5‐month‐old and 8‐month‐old 5xFAD mice (Figure S3). In addition, to further confirm the observation that neuron loss was restricted to the core area of Aβ deposits, we repeated the procedures described above using 12‐month‐old APPswe/PSEN1dE9 mice (another mouse model of Aβ deposition) (Figure S4). The results of these experiments showed that NeuN‐positive neuron loss was restricted to the core area of both large and small Aβ deposits in 12‐month‐old APPswe/PSEN1dE9 mice (Figure S4).

Overall, these results indicate that NeuN‐positive neuron loss is restricted to the core areas of Aβ deposits.

### 2.2 NeuN‐positive neuron loss is correlated with the size of Aβ deposits

**FIGURE 2:**
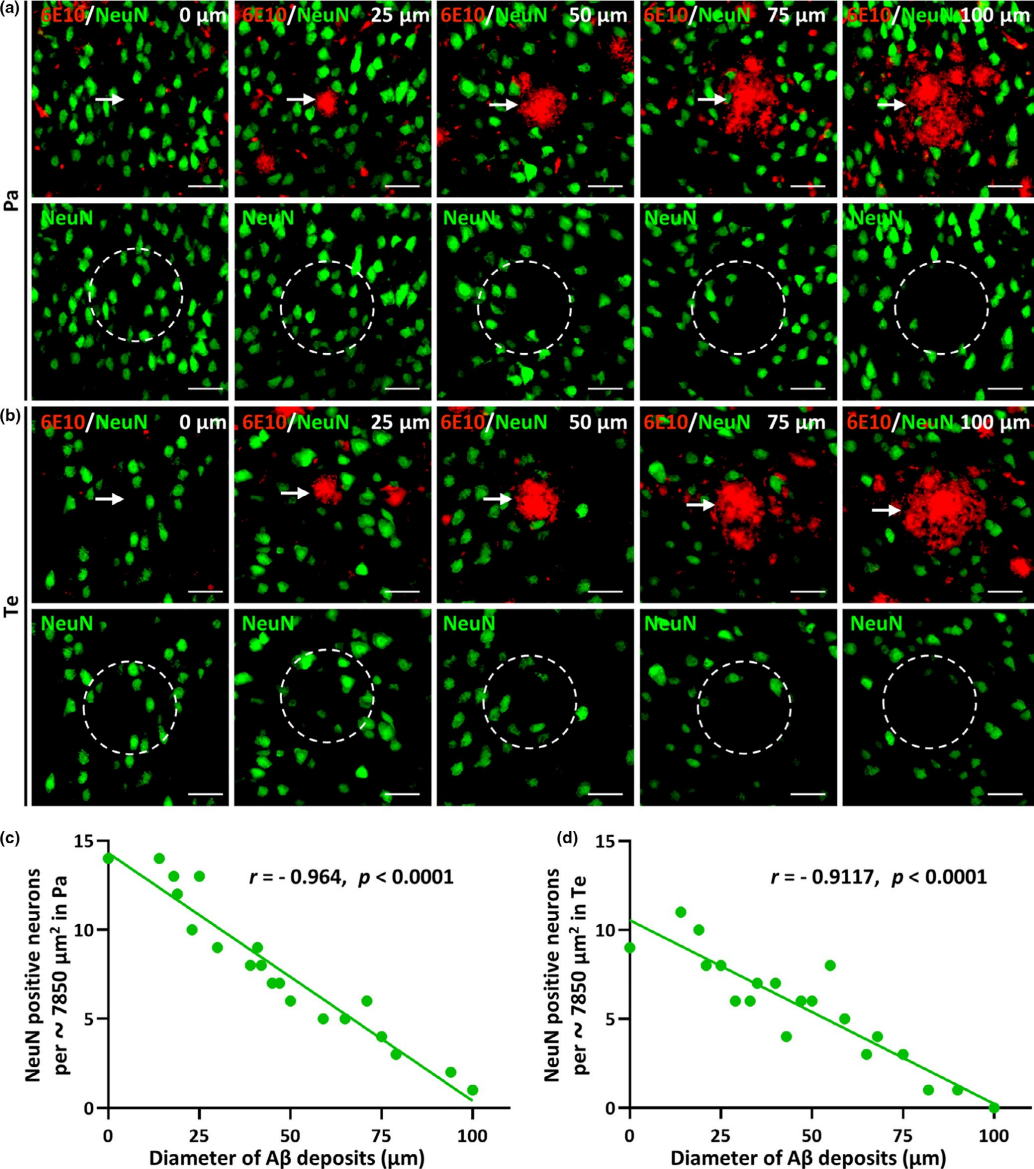
NeuN‐positive neuronal loss is correlated with the size of Aβ deposits. (a and b) Representative high‐magnification micrographs of the parietal (Pa) and temporal cortex (Te), respectively, from 12‐month‐old 5xFAD mice with co‐labeling of NeuN‐positive neurons (green) and Aβ deposits (red). The arrows indicate the Aβ deposits. The scale bars were 40 µm for all panels. Representative Aβ deposits of different sizes (approximately 0, 25, 50, 75, or 100 µm in diameter) in the Pa or Te of 5xFAD mice are exhibited and outlined with a 100 µm diameter circle. The number of NeuN‐positive neurons in each of the dotted circles was counted for quantitative analysis. (c and d) The correlations between the number of NeuN‐positive neurons in the dotted circles (per approximately 7850 µm^2^) and the diameter of Aβ deposits in the Pa or Te, respectively, in 5xFAD mice (*r* = −0.964, *p* < 0.0001 in Pa, *r* = −0.9117, *p* < 0.0001 in Te). Data derived from twenty images of four different mice

To determine whether the degree of NeuN‐positive neuron loss is correlated with the size of Aβ deposits, we counted the number of NeuN‐positive neurons in the core area of Aβ deposits of different sizes in the Pa (Figure 2a) and Te (Figure 2b) of 5xFAD mice. In the Pa and Te, the number of NeuN‐positive neurons was negatively correlated with the size of the Aβ deposits (Figure 2c, *r* = −0.964, *p* < 0.0001; Figure 2d, *r* = −0.9117, *p* < 0.0001).

### 2.3 Neurofilament heavy (NF‐H) positive axon loss and axonal pathology are restricted to the core area of Aβ deposits

**FIGURE 3:**
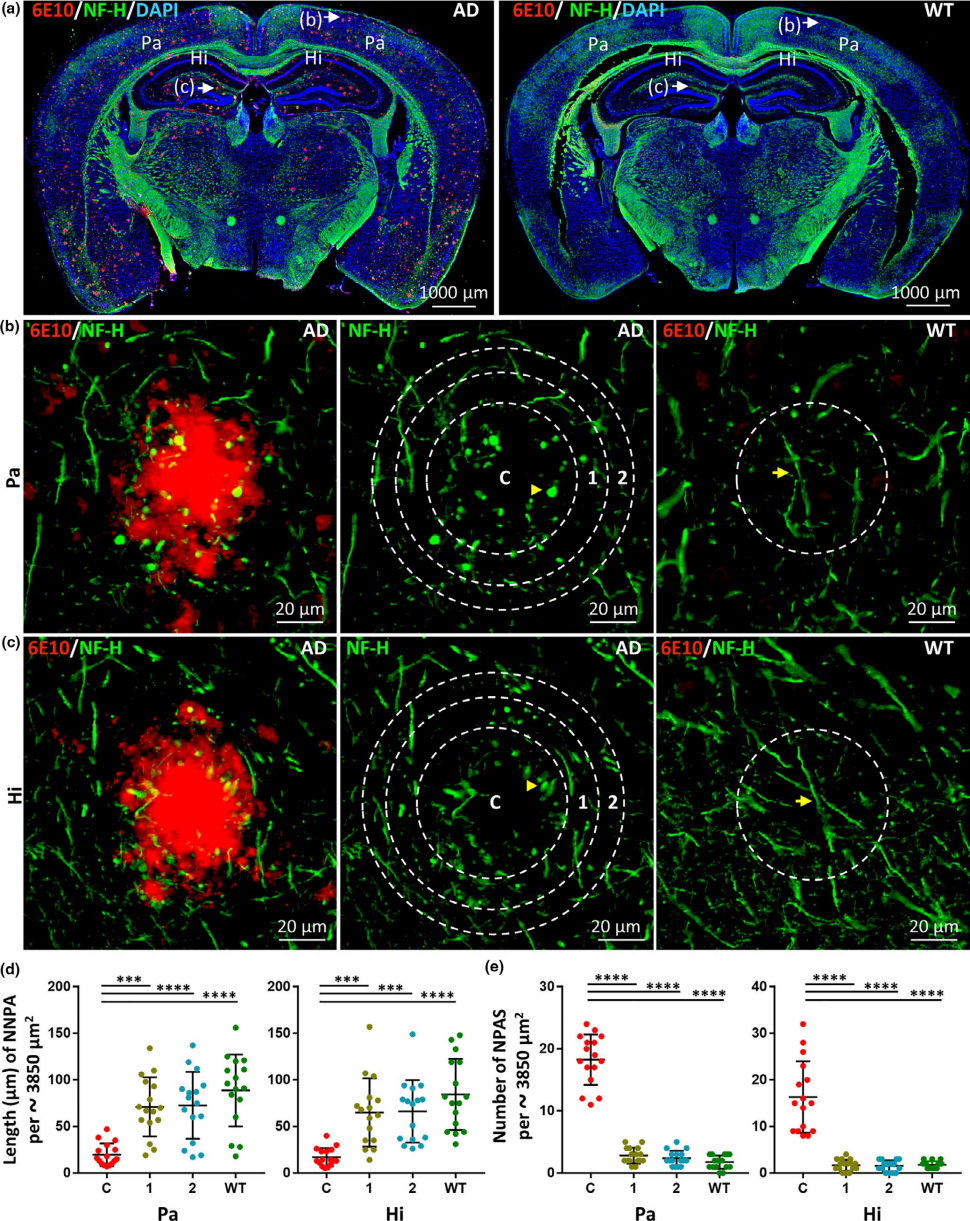
NF‐H‐positive axonal loss and axonal pathology are restricted to the core area of Aβ deposits. (a) Representative images of double‐label immunofluorescence staining of coronal brain sections from 12‐month‐old 5xFAD and WT mice. Aβ deposits labeled with anti‐Aβ antibody (6E10) and Alexa Fluor 647 goat anti‐mouse secondary antibody are shown in red. NF‐H‐positive axons labeled with anti‐NF‐H antibody and Alexa Fluor 488 goat anti‐rabbit secondary antibody are shown in green. Nuclei counterstained with DAPI are shown in blue. Scale bars, 1000 µm. Representative images of Aβ deposits in the parietal cortex (Pa) and hippocampus (Hi) were taken with high magnification (shown in b and c, respectively). The core Aβ deposit circle (C) is surrounded by two adjacent concentric rings (1, 2) with the same area. The corresponding circular regions are shown in WT mice. In comparison with WT mice, normal NF‐H‐positive axons (NNPA, see yellow arrow) were significantly shortened within the core circle of Aβ deposits in 5xFAD mice, which also showed abnormal NF‐H‐positive axonal spheroids (NPAS, see yellow arrowhead). Scale bars, 20 µm. (d) Quantitative analysis of the total length of NNPA in the Pa and Hi in 5xFAD and WT mice. Quantification of the total length of NNPA (per approximately 3850 µm^2^) indicated a significant loss of NF‐H‐positive axons in the core of Aβ deposits. Data derived from ‐sixteen images of eight different mice for each group (*n* = 16). Data represent mean ±SD. ***p < 0.001; ****p < 0.0001. (e) Quantitative analysis of the number of NPAS formed in the Pa and Hi in 5xFAD and WT mice. The numbers of NPAS (per approximately 3850 µm^2^) in the core circle (C) and two adjacent concentric rings (1, 2) of 5xFAD mice and the corresponding circular region of WT mice were counted and compared. The number of NPAS in the core circle of Aβ deposits in 5xFAD mice was increased significantly in comparison with that of the adjacent regions and the corresponding region in WT mice. Data derived from sixteen images of eight different mice for each group (*n* = 16). Data represent mean ±SD. ****p < 0.0001

To assess the effects of endogenous Aβ deposits on axons, we compared the brains of 5xFAD and WT mice using double immunofluorescence staining. Monoclonal anti‐Aβ antibodies (6E10) that recognize Aβ deposits (red) were used in conjunction with anti‐NF‐H antibodies to label axons (green) (Figure 3). The length of normal NF‐H‐positive axons (NNPA) in the core area of Aβ deposits in the Pa and hippocampus (Hi) was significantly decreased in comparison with the two adjacent areas in 5xFAD mice and the corresponding area of age‐matched WT mice (Figure 3b‐d, in Pa, C vs. 1, *p* < 0.001; C vs. 2, *p* < 0.0001; C vs. WT, *p* < 0.0001; in Hi, C vs. 1, *p* < 0.001; C vs. 2, *p* < 0.001; C vs. WT, *p* < 0.0001). There were no significant differences in the length of NNPA between the areas adjacent to the Aβ deposits in 5xFAD mice and the corresponding area of age‐matched WT mice (Figure 3b‐d, 1 vs. WT, *p* > 0.05; 2 vs. WT, *p* > 0.05; 1 vs. 2, *p* > 0.05). The number of abnormal NF‐H‐positive axonal spheroids (NPAS) in the core area of Aβ deposits in the Pa and Hi was significantly increased in comparison with that of the two adjacent areas in 5xFAD mice, as well as significantly increased in comparison with number of NPAS in the corresponding area of age‐matched WT mice (Figure 3b, c, and e, both in Pa and Hi, C vs. 1, *p* < 0.0001; C vs. 2, *p* < 0.0001; C vs. WT, *p* < 0.0001). No significant difference was identified between the number of NPAS in the areas adjacent to Aβ deposits in 5xFAD mice and the number of NPAS in the corresponding area of age‐matched WT mice (Figure 3 b, c, and e, both in Pa and Hi, 1 vs. WT, *p* > 0.05; 2 vs. WT, *p* > 0.05; 1 vs. 2, *p* > 0.05). These results indicate that NF‐H‐positive axon loss and axonal pathology are restricted to the core area of Aβ deposits.

### 2.4 NF‐H‐positive axon loss and axonal pathology are correlated with the size of Aβ deposits

**FIGURE 4:**
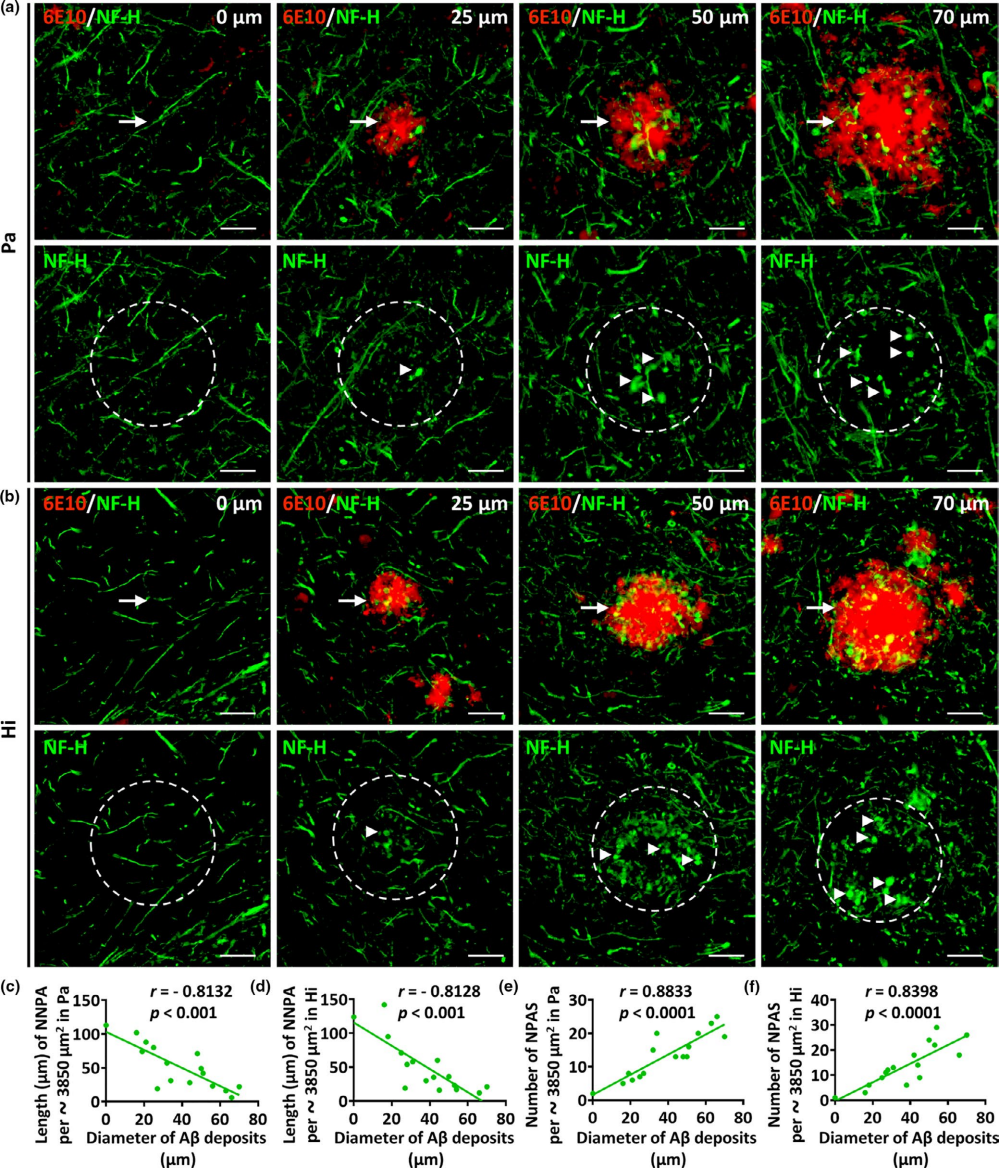
NF‐H‐positive axonal loss and axonal pathology are correlated with the size of Aβ deposits. (a and b) Representative micrographs with high magnification of the parietal cortex (Pa) and hippocampus (Hi) from 12‐month‐old 5xFAD mice co‐labeled to show NF‐H (green) and Aβ deposits (red). Aβ deposits of different sizes (approximately 0, 25, 50, 70 µm in diameter) in the Pa and Hi of 5xFAD mice were selected and exhibited. Dotted circles of approximately 3850 µm^2^ in area were outlined centered on the Aβ deposits. The arrows indicate the Aβ deposits and the arrowheads indicate abnormal NF‐H‐positive axonal spheroids (NPAS). The number of NPAS and the length of normal NF‐H‐positive axons (NNPA) in the dotted circles were determined for quantitative analysis. The scale bars were 20 µm for all panels. (c and d) Correlations between the length of NNPA in the area of the dotted circles (per approximately 3850 µm^2^) and the size of Aβ deposits in the Pa or Hi, respectively, of 5xFAD mice are shown (*r* = ‐ 0.8132, *p* < 0.001 in Pa, *r* = −0.8128, *p* < 0.001 in Hi). (e and f) Correlations between the number of NPAS in the area of the dotted circles (per approximately 3850 µm^2^) and the size of Aβ deposits in the Pa or Hi, respectively, of 5xFAD mice are shown (*r* = 0.8833, *p* < 0.0001 in Pa, *r* = 0.8398, *p* < 0.0001 in Hi). Data were derived from sixteen images of four different mice

To determine whether NF‐H‐positive axon loss is correlated with Aβ deposit size, we counted the total length of NNPA in the core area of different sizes of Aβ deposits in the Pa (Figure 4a) and Hi (Figure 4b) of 5xFAD mice. The length of NNPA was negatively correlated with the size of Aβ deposits (Figure 4c, *r* = −0.8132, *p* < 0.001; Figure 4d, *r* = −0.8128, *p* < 0.001). The number of NPAS in the core area of Aβ deposits in the Pa and Hi of 5xFAD mice was positively correlated with the size of Aβ deposits (Figure 4e, *r* = 0.8833, *p* < 0.0001; Figure 4f, *r* = 0.8398, *p* < 0.0001).

### 2.5 Myelin basic protein (MBP) positive myelin sheath loss and myelinic pathology are restricted to the core area of Aβ deposits

**FIGURE 5:**
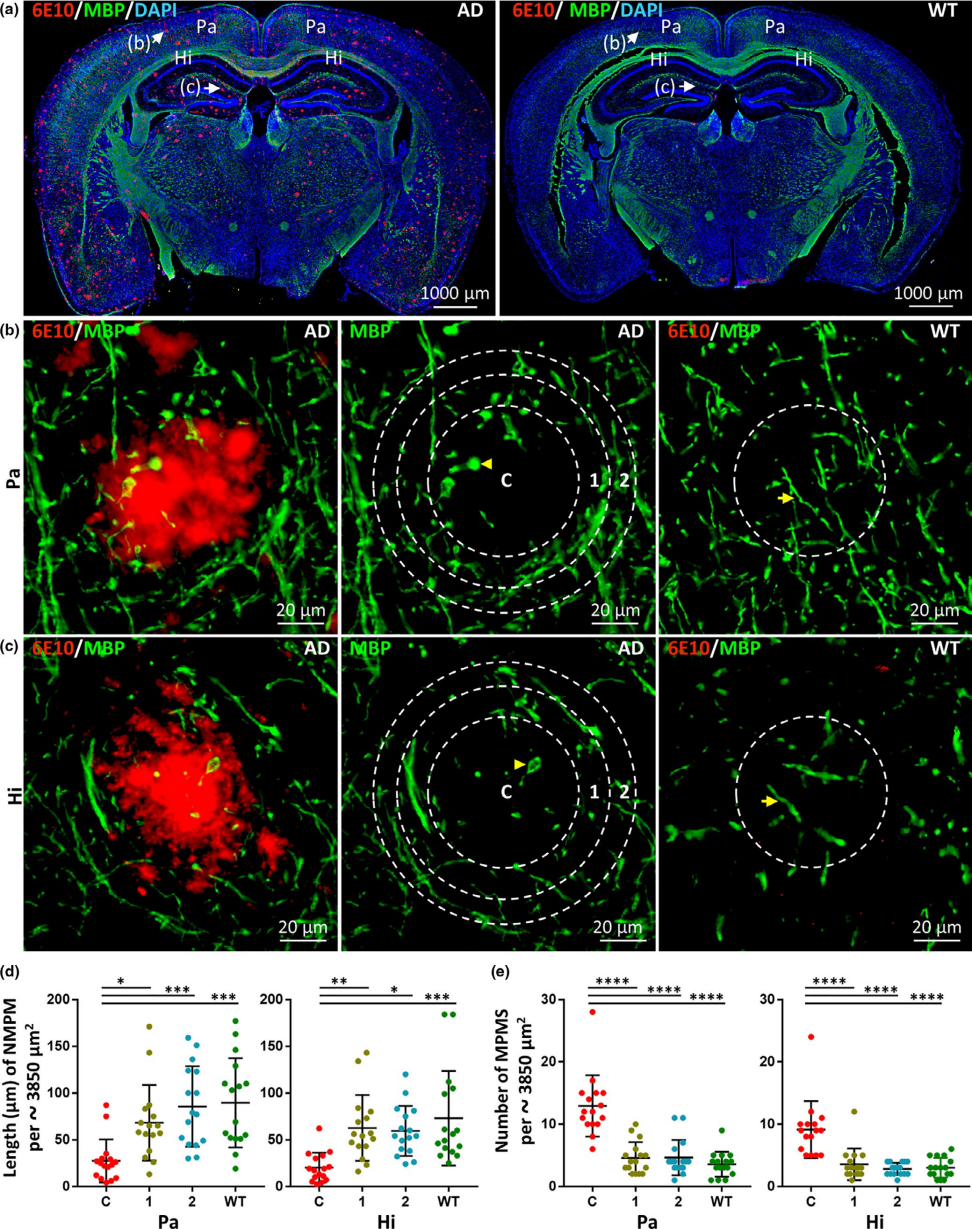
MBP‐positive myelin sheath loss and myelinic pathology are restricted to the core area of Aβ deposits. (a) Representative images of double‐label immunofluorescence staining of coronal brain sections from 12‐month‐old 5xFAD and WT mice. Aβ deposits labeled with anti‐Aβ antibody (6E10) and Alexa Fluor 647 goat anti‐mouse secondary antibody are shown in red. MBP‐positive myelin labeled with anti‐MBP antibody and Alexa Fluor 488 goat anti‐rabbit secondary antibody is shown in green. Nuclei counterstained with DAPI are shown in blue. Scale bars, 1000 µm. Representative images of Aβ deposits in the parietal cortex (Pa) and hippocampus (Hi) were taken with high magnification (shown in b and c, respectively). Aβ deposits in the Pa and Hi of 5xFAD mice are shown and outlined as a core circle (C) surrounded by two adjacent concentric rings (1, 2) with the same area. The corresponding circular regions are shown in WT mice. Normal MBP‐positive myelin (NMPM, see yellow arrow) decreased significantly and abnormal MBP‐positive myelin spheroids (MPMS, see yellow arrowhead) formed in the core circle of Aβ deposits in 5xFAD mice. Scale bars, 20 µm. (d) Quantitative analysis of the total length of NMPM (per approximately 3850 µm^2^) in the Pa and Hi in 5xFAD and WT mice. In comparison to the circular region of WT mice and the two adjacent concentric rings (1, 2) in 5xFAD mice, a significant loss of NMPM was detected in the core area of Aβ deposits. Data derived from eight images of four different mice for each group (*n* = 8). Data represent mean ±SD. *p < 0.05; **p < 0.01; ***p < 0.001‐. (e) Quantitative analysis of the number of MPMS (per approximately 3850 µm^2^) formed in the Pa and Hi in 5xFAD and WT mice. The numbers of MPMS in the core circle (C) and two adjacent concentric rings (1, 2) of 5xFAD mice and the corresponding circular region of WT mice (labeled as WT) were compared. The number of MPMS in the core circle of Aβ deposits in 5xFAD mice was significantly increased in comparison with the adjacent areas in 5xFAD mice and the corresponding area in WT mice. Data derived from sixteen images of eight different mice for each group (*n* = 8). Data represent mean ±*SD*. ****p < 0.0001

To evaluate the effects of endogenous Aβ deposits on the myelin sheath, we compared the brains of 5xFAD and WT mice using double immunofluorescence staining. Monoclonal anti‐Aβ antibodies (6E10) that recognize Aβ deposits (red) were used in conjunction with anti‐MBP antibodies to label myelin (green) (Figure 5). The length of normal MBP‐positive myelin sheaths (NMPM) in the core area of Aβ deposits in the Pa and Hi was significantly decreased in comparison with the two adjacent areas in 5xFAD mice and the corresponding area in age‐matched WT mice (Figure 5b‐d, in Pa, C vs. 1, *p* < 0.05; C vs. 2, *p* < 0.001; C vs. WT, *p* < 0.001; in Hi, C vs. 1, *p* < 0.01; C vs. 2, *p* < 0.05; C vs. WT, *p* < 0.001). The NMPM length was not significantly different in the areas adjacent to Aβ deposits in 5xFAD mice and in the corresponding area of age‐matched WT mice (Figure 5b‐d, both in Pa and Hi, 1 vs. WT, *p* > 0.05; 2 vs. WT, *p* > 0.05; 1 vs. 2, *p* > 0.05). The number of abnormal MBP‐positive myelin spheroids (MPMS) in the core area of Aβ deposits in the Pa and Hi was significantly increased in comparison with that of the two adjacent areas in 5xFAD mice and that of the corresponding area of age‐matched WT mice (Figure 5b, c, and e, in Pa, C vs. 1, *p* < 0.0001; C vs. 2, *p* < 0.0001; C vs. WT, *p* < 0.0001; in Hi, C vs. 1, *p* < 0.0001; C vs. 2, *p* < 0.0001; C vs. WT, *p* < 0.0001). There were no differences in the number of MPMS between the areas adjacent to the Aβ deposits in 5xFAD mice and the corresponding areas of age‐matched WT mice (Figure 5 b, c, and e, both in Pa and Hi, 1 vs. WT, *p* > 0.05; 2 vs. WT, *p* > 0.05; 1 vs. 2, *p* > 0.05). These results indicate that MBP‐positive myelin sheath loss and myelinic pathology are restricted to the core area of Aβ deposits.

### 2.6 MBP‐positive myelin sheath loss and myelinic pathology are correlated with the size of Aβ deposits

**FIGURE 6:**
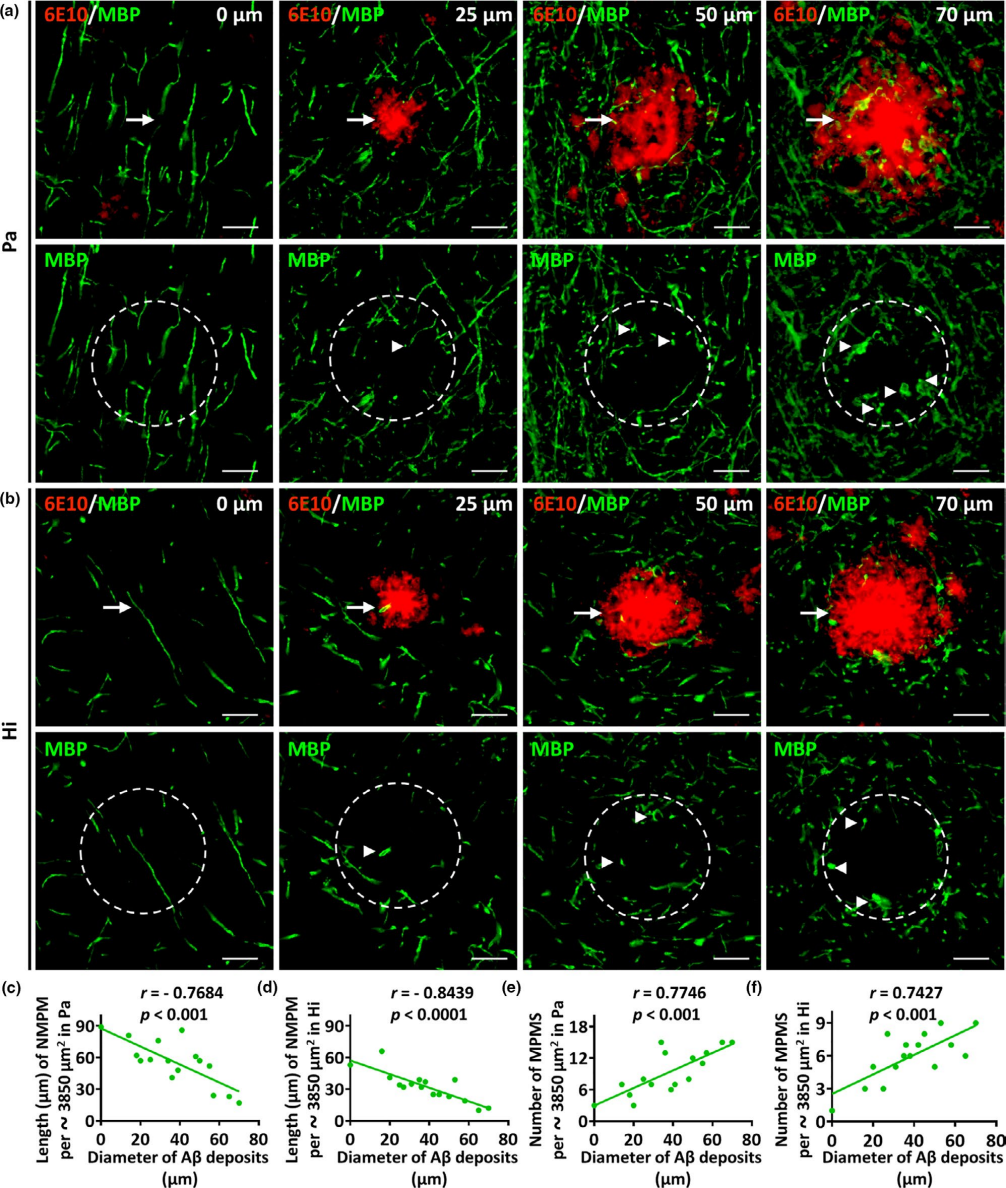
MBP‐positive myelin sheath loss and myelinic pathology are correlated with the size of Aβ deposits. (a and b) Representative micrographs of the parietal cortex (Pa) and hippocampus (Hi) from 12‐month‐old 5xFAD mice co‐labeled with MBP (green) and Aβ deposits (red) at high magnification. Aβ deposits of different sizes (approximately 0, 25, 50, and 70 µm in diameter) were selected and exhibited. Dotted circles of approximately 3850 µm^2^ in area were outlined centered on the Aβ deposits. The arrows indicate the Aβ deposits and the arrowheads indicate abnormal MBP‐positive myelin spheroids (MPMS). The number of MPMS and the length of normal MBP‐positive myelin (NMPM) in dotted circles were determined for quantitative analysis. The scale bars are 20 µm for all panels. (c and d) The correlations between the length of NMPM in the dotted circles (per approximately 3850 µm^2^) and the size of Aβ deposits in the Pa or Hi of 5xFAD mice are shown (*r* = −0.7684, *p* < 0.001 in Pa, *r* = −0.8439, *p* < 0.0001 in Hi). (e and f) The correlations between the number of NPAS in the dotted circles (per approximately 3850 µm^2^) and the size of Aβ deposits in the Pa or Hi of 5xFAD mice are shown (*r* = 0.7746, *p* < 0.001 in Pa, *r* = 0.7427, *p* < 0.001 in Hi). Data were derived from sixteen images of four different mice

To determine whether MBP‐positive myelin sheath loss is correlated with Aβ deposit size, we counted the total length of NMPM in the core area of Aβ deposits in the Pa (Figure 6a) and Hi (Figure 6b) of 5xFAD mice. In the Pa and Hi, the length of NMPM was negatively correlated with Aβ deposit size (Figure 6c, *r* = −0.7684, *p* < 0.001; Figure 6d, *r* = −0.8439, *p* < 0.0001). The number of MPMS in the core areas of Aβ deposits of different sizes in the Pa and Hi of 5xFAD mice was positively correlated with the size of Aβ deposits (Figure 6a and e, *r* = 0.7746, *p* < 0.001; Figure 6b and f, *r* = 0.7427, *p* < 0.001).

### 2.7 IBA‐1‐positive microglia are highly clustered in the core rather than the periphery of Aβ deposits

**FIGURE 7:**
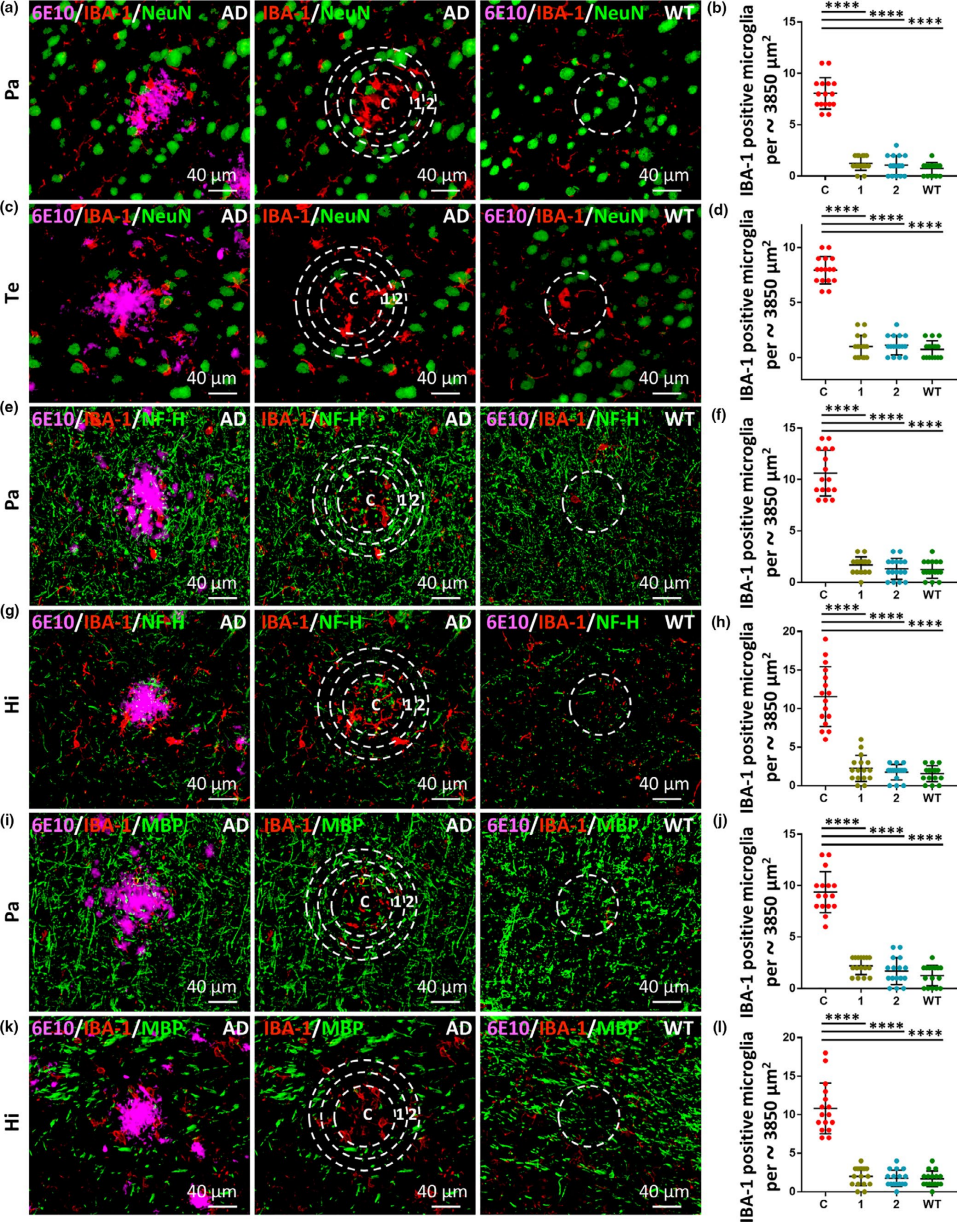
IBA‐1‐positive microglia are highly clustered in the core of Aβ deposits rather than the periphery. (a and c) Representative images of triple‐label immunofluorescence staining in the parietal cortex (Pa) and temporal cortex (Te), respectively, from 12‐month‐old 5xFAD and WT mice, labeled with anti‐Aβ antibody 6E10 (magenta, a marker for Aβ deposits), anti‐IBA‐1 antibody (red, a marker for microglia) and anti‐NeuN antibody (green, a marker for neuron bodies). Dotted core circles (C) with two adjacent concentric rings (1, 2) of the same area (approximately 3850 µm^2^ in area) are outlined centered on the Aβ deposits (approximately 70 µm in diameter) in the Pa and Te, respectively, in 5xFAD mice. Circular regions corresponding to the same areas are shown in WT mice. Scale bars, 40 µm. (b and d) Quantitative analyses of the number of IBA‐1‐positive microglia present in the core circle (labeled as C) and two adjacent concentric rings (labeled as 1, 2) of 5xFAD mice and the corresponding circular region of WT mice (labeled as WT) are shown. IBA‐1‐positive microglia were highly clustered in the core of Aβ deposits. 6E10 (magenta)/IBA‐1 (red)/NF‐H (green, a marker for axons) triple‐label immunofluorescence staining (e and g) and quantification of IBA‐1‐positive microglia (f and h) were performed to examine the activation and distribution of IBA‐1 in the Pa or hippocampus (Hi) sections containing Aβ deposits and axonal pathology, respectively. 6E10 (magenta)/IBA‐1 (red)/MBP (green, a marker for myelin sheaths) triple‐label immunofluorescence staining (i and k) and quantification of IBA‐1‐positive microglia (j and l) were performed to examine the activation and distribution of IBA‐1 in the Pa or Hi sections containing Aβ deposits and myelinic pathology, respectively. Data were derived from sixteen images of eight different mice for each group (*n* = 16). Data represent mean ±SD. ****p < 0.0001

The results described above show that neuronal loss was restricted to the core rather than the peripheral area of Aβ deposits. Next, experiments were conducted to explain the mechanism underlying these findings. Recent convincing evidence suggests that microglia modulate neuronal loss in AD and other neurodegenerative diseases (Bartels et al., 2020), and Aβ deposits commonly induce microgliosis in AD (Selkoe & Hardy, 2016). Therefore, we proposed that Aβ deposits attracted microglia to cluster in the core area rather than the peripheral area of Aβ deposits, and thus Aβ toxicity and microgliosis‐induced neuroinflammation toxicity were both restrained in the core area. As a result, neuronal loss (including loss of the neuron body, axon, and myelin sheath) occurred in the core area rather than the peripheral area of Aβ deposits. To test this proposal, we performed triple‐label immunofluorescence staining (6E10/IBA‐1/NeuN or/NF‐H or/MBP) using brain slices from 12‐month‐old 5xFAD mice and age‐matched WT mice (Figure 7). The number of IBA‐1‐positive microglia in the core area of Aβ deposits in the brain (including the Pa, TE, and Hi regions) was significantly increased in comparison with that of the two adjacent areas in 5xFAD mice, as well as significantly increased in comparison with the number of microglia in the corresponding area of age‐matched WT mice (Figure 7, all in Pa, Te and Hi, C vs. 1, *p* < 0.0001; C vs. 2, *p* < 0.0001; C vs. WT, *p* < 0.0001). No significant difference was identified between the number of microglia in the areas adjacent to Aβ deposits in 5xFAD mice and the number of microglia in the corresponding area of age‐matched WT mice (Figure 7, all in Pa, Te and Hi, 1 vs. WT, *p* > 0.05; 2 vs. WT, *p* > 0.05; 1 vs. 2, *p* > 0.05). These results indicate that microgliosis was restricted to the core area of Aβ deposits. By considering these results together with the finding that the losses of the neuron body, axon, and myelin sheath were restricted to the core area of Aβ deposits (Figures 1, 3, and 5), we conclude that the Aβ deposits attracted the microglia to cluster in the core area where Aβ toxicity and neuroinflammation toxicity were both restrained, and this mechanism can probably explain why neuronal loss occurred in the core area of Aβ deposits rather than the peripheral area.

## 3 DISCUSSION

Despite years of study focused on the relationship between Aβ deposits and neuronal loss, this relationship remains unclear. Some researchers believe that Aβ deposits are not closely associated with neuronal loss or brain atrophy, while others argue that Aβ deposits have a close relationship with both of these processes (Chetelat, 2013; Giacobini & Gold, 2013; Hardy & Higgins, 1992; Herrup, 2015). One of the main factors causing this discrepancy is the confounding presence of pathologic tau. It is well established that pathologic tau is strongly correlated with neurodegeneration in AD patients, indicating that pathologic tau contributes to neuronal loss and brain atrophy (La Joie et al., 2020; Xia et al., 2017). In this study, we used the 5xFAD mouse model, which develops abundant Aβ deposits without accumulating pathologic tau in the aged brain. Thus, we analyzed the relationship between Aβ deposits and neuronal loss without the effects of pathologic tau.

NeuN is a neuronal nuclear antigen commonly used as a marker for neurons (Herculano‐Houzel & Lent, 2005). The anti‐NeuN antibody specifically recognizes the nucleus and cytoplasm of a vast majority of neurons. We found that NeuN‐positive neurons were lost in the core area of Aβ deposits, but not in adjacent areas, in the brains of 5xFAD mice at different ages and another aged Aβ mice model (APPswe/PSEN1dE9). Considering that neuronal loss in the core area of Aβ deposits may be due to Aβ toxicity or growth of deposits to replace the space previously used by neurons, we analyzed the number of Cyt C‐positive aggregates (marker of cellular death) and the intensity of MAP2 (marker of neurodegeneration) in the core area of Aβ deposits, and our results clearly indicate cellular death and neurodegeneration in the core area of Aβ deposits. These data suggest that endogenous Aβ deposits are toxic to neurons in the immediate area of the deposits, but not widely toxic for neurons in adjacent areas. Researchers previously reported that neurons were macroscopically lost in the Layer 5 cortex of aged 5xFAD mice (Buskila et al., 2013). Here we focused on the microscopic level of Aβ deposits, and found that neuronal loss occurred mainly in the core area of Aβ deposits, but not in adjacent areas, in the cerebral cortex (including the Pa and Te). Although the exact mechanisms through which Aβ accumulation induces neuronal toxicity are unclear, inflammation, oxidative stress, apoptosis, and mitochondrial and synaptic dysfunction are potential mechanisms that might have caused neuronal loss in the core area of Aβ deposits in our study (Carrillo‐Mora et al., 2014; Rajasekhar et al., 2015; Reiss et al., 2018). In agreement with our results, Eimer and Vassar reported that neuronal loss is correlated with intraneuronal Aβ42 accumulation and Caspase‐3 activation in 5xFAD mice (W. Eimer & Vassar, 2013). Here we expand on these findings and show that neuronal loss is significantly correlated with the size of cerebral Aβ deposits.

NeuN antibodies recognize the cell body of neurons, but not the axon and myelin sheath of neurons. NF‐H is one of the major components of the axon and is used as a marker for axons. MBP is one of the major components of the myelin sheath and is widely used as a myelin marker. Previous studies demonstrated axon and myelin pathologies in the brains of AD patients and AD model mice (Blazquez‐Llorca et al., 2017; Schmued et al., 2013; Xiao et al., 2011; Zhan et al., 2015). Here, we focused on axon and myelin pathologies in the areas of cerebral Aβ deposits. We found that both NF‐H‐positive axons and MBP‐positive myelin sheaths were lost in the core area of Aβ deposits, but not in adjacent areas, in the Pa and Hi of the brains of aged 5xFAD mice. Meanwhile, abundant abnormal axonal spheroids and myelin spheroids occurred in the core area of Aβ deposits, but not in adjacent areas. These data indicate that endogenous Aβ deposits are toxic to axons and the myelin sheath in the direct area of deposition, but not widely toxic in adjacent areas. Although the exact mechanisms through which Aβ accumulation is toxic to axons and the myelin sheath are unclear, possible explanations for this toxicity include inflammation, oxidative stress, and iron overload (Papuc & Rejdak, 2020; Salvadores et al., 2017). Previous findings concerning the relationship between Aβ deposits and axons or the myelin sheath are controversial. While some researchers reported that Aβ deposits were correlated well with losses of axons or the myelin sheath (Dean et al., 2017; Kaya et al., 2020; Marin et al., 2016; van Westen et al., 2016), other studies indicated that Aβ deposits were not correlated with the loss of axons or the myelin sheath (Adalbert et al., 2009; McAleese et al., 2015; Rutten‐Jacobs et al., 2011; Zetterberg et al., 2016). The toxic effects of pathologic tau on axons and the myelin sheath may account for some of the contradictory findings about the relationship between Aβ deposits and axons or the myelin sheath. In this study, the effects of pathologic tau were ruled out. Both NNPA and NMPM loss were significantly correlated with the size of cerebral Aβ deposits. Furthermore, the number of abnormal NPAS and MPMS correlated with the size of cerebral Aβ deposits.

In addition to our finding that neuronal loss was restricted to the core area of Aβ deposits, our results clearly show that the abundance of IBA‐1 positive microglia was increased in the core area of Aβ deposits, but not in continuous adjacent areas. These results suggest that the increased abundance of microglia restrained Aβ toxicity to the core area together with microglia‐induced neuroinflammation, and this finding probably explains why neuronal loss occurred in the core area rather than the peripheral area of Aβ deposits. Our finding is consistent with a previous study showing that activated microglia play a critical role in neurodegeneration in AD and act downstream of Aβ deposits to mediate neuronal loss (Spangenberg et al., 2016).

Previous reports (Serrano‐Pozo et al., 2011; Walker, 2020) have indicated that the diverse morphologies of Aβ plaques in AD patients include classical dense‐cored plaques, core‐space‐corona pattern plaques, and a range of diffuse Aβ plaques of different sizes and shapes. Classical dense‐cored Aβ plaques are abundant in 5xFAD mice and were analyzed in this study. However, core‐space‐corona pattern plaques are rare in 5xFAD mice. Therefore, future experiments should assess the toxicity of core‐space‐corona pattern plaques and other types of plaques, as well as their ability to recruit glial cells.

In summary, we clearly showed that significant neuronal loss (including neuron, axon, and myelin sheath loss) was restricted to the core areas of cerebral Aβ deposits and did not occur in adjacent areas. This restricted neuronal loss was probably due to the attraction of microglia to the core area of Aβ deposits, where they formed clusters associated with locally restrained Aβ toxicity and neuroinflammation toxicity. In addition, we showed that neuronal loss was significantly correlated with the size of cerebral Aβ deposits.

## 4 EXPERIMENTAL PROCEDURES

### 4.1 Animals

Animal experiments were performed in compliance with the Health Guide for the Care and Use of Laboratory Animals at Capital Medical University. The protocols were approved by the Animal Ethics Committee at Capital Medical University, Beijing, China. 5xFAD mice (Tg6799, MMRRC Stock No: 34840‐JAX) and APPswe/PSEN1dE9 mice (MMRRC Stock No: 34832‐JAX) were obtained from The Jackson Laboratory, the 5xFAD mice were maintained in a breeding colony with C57BL/6 mice. Genotyping was performed by PCR analysis with tail‐tip DNA according to Jackson Laboratory's instructions. The sex of 5xFAD mice and WT mice at different ages used in this study was as follows: four females and four males at the age of around 12 months, two females and one male at the age of 8 months, one female and two males at the age of 5 months. In addition, one female and two male APPswe/PSEN1dE9 mice and WT mice at the age of 12 months were used in this study.

### 4.2 Perfusion and tissue processing

Mice were euthanized by a lethal intraperitoneal injection of sodium pentobarbital and perfused intracardially with phosphate‐buffered saline (PBS, 0.1 M; pH 7.4) followed by 50 mL of ice‐cold 4% paraformaldehyde (PFA, Solarbio) in PBS. Brain tissue samples were dissected and post‐fixed in 4% PFA at 4°C overnight. Fixed brain tissue samples were embedded in paraffin and coronally sectioned into 4 μm‐thick slices for later use.

### 4.3 Immunofluorescence staining of mouse brain sections

The brain sections were deparaffined and subjected to microwave‐mediated antigen retrieval, followed by treatment with 3% (v/v) H_2_O_2_ in methanol (Sigma‐Aldrich) for 10 min to quench endogenous peroxidase activity. After three washes with PBS, the sections were incubated in 5% (w/v) bovine serum albumin (BSA, Sigma‐Aldrich) for 20 min at room temperature and subsequently incubated at 4°C overnight with phospho‐tau (S202, T205) monoclonal antibody (AT8, 1:200, MN1020, Thermo Fisher) or anti‐tau (phospho S396) antibody (1:1000, ab109390, Abcam) to verify the absence of phospho‐tau. A goat polyclonal secondary antibody to rabbit IgG (H&L Alexa Fluor 488, 1:3000, ab150077, Abcam) or a goat polyclonal secondary antibody to mouse IgG (H&L Alexa Fluor 647, 1:3000, ab150115, Abcam) was used as the secondary antibody. For co‐localization of Aβ deposits and neurons, 2‐step double‐labeling immunofluorescence staining was performed. Briefly, the sections were incubated with the anti‐Aβ antibody (6E10, 1:400, 803015, BioLegend) at 4°C overnight, followed by a goat anti‐mouse secondary antibody, as described above. The sections were delipidated in acetone for 90 seconds prior to three washes in PBS. The delipidated sections were incubated with anti‐NeuN antibody (1:1000, ab177487, Abcam), anti‐MBP antibody (1:200, ab40390, Abcam), anti‐NF‐H antibody (1:1000, ab8135, Abcam), anti‐Cy C antibody (1:100, ab133504, Abcam), or anti‐MAP2 antibody (1:200, ab183830, Abcam) at 4°C overnight, followed by a goat anti‐rabbit secondary antibody, as described above. Triple‐labeling immunofluorescence staining was performed sequentially by probing one antigen after another to examine the distribution of three different antigens in the same brain region. The primary antibodies were from three different hosts and consisted of a mouse monoclonal antibody against Aβ (6E10, 1:400, 803015, BioLegend), a goat polyclonal antibody against Iba1 (IBA‐1, 1:1000, 011–27991, Wako), and rabbit polyclonal antibodies against NeuN, NF‐H, or MBP. Double‐ or triple‐labeled immunofluorescence stained sections were placed in mounting medium with DAPI to preserve fluorescence. All images were acquired by whole‐slide scanning with a Pannoramic MIDI digital slide scanner (3DHISTECH, Hungary).

### 4.4 Image analysis

Sections from 5xFAD and WT mice at 12 months old (*n* = 8 in each group), 8 months old (*n* = 3 in each group), and 5 months old (*n* = 3 in each group), as well as from APPswe/PSEN1dE9 and WT mice at 12 months old (*n* = 3 in each group), were labeled with 6E10 (red) antibodies and co‐labeled with neuron‐associated antibodies, as described above. Images were viewed and taken by the Pannoramic Viewer with seamless zooming. For the image analysis, the red channel (6E10) and green channel (NeuN, NF‐H, MBP, Cyt C, MAP2, IBA‐1) were opened separately. The edges of the area of red fluorescence were outlined and marked as a circle to determine the area occupied by the Aβ deposits, and two concentric circles of the same area were outlined surrounding the core circle of Aβ deposits. The number of NeuN‐positive neurons, number of NPAS, number of MPMS, length of NNPA, length of NMPM, number of Cyt C‐positive aggregates, intensity of MAP2, and number of IBA‐1‐positive microglia in the core circle (C), concentric circle 1 (1) and concentric circle 2 (2) of the indicated mice were determined.

### 4.5 Statistical analysis

The GraphPad Prism 8.0 software package was used for the statistical analysis. The ordinary one‐way ANOVA with multiple comparisons testing was used to assess the statistical significance of differences in group means (Lee et al., 2021). The data are presented in the bar graphs as mean ±SD. Significant differences were considered at *p < 0.05, **p < 0.01, ***p < 0.001 and ****p < 0.0001. The correlations between the size of Aβ deposits and the number of NeuN‐positive neurons, number of NPAS, number of MPMS, length of NNPA, length of NMPM, number of Cyt C‐positive aggregates, intensity of MAP2, or number of IBA‐1‐positive microglia, were investigated using Pearson correlation coefficients (*r*) with *p* values to assess the strength of the correlation.

## CONFLICT OF INTEREST

None declared.

## AUTHOR CONTRIBUTIONS

BC, JZ, NW, and JL designed the study. BC and JZ analyzed the data and wrote the manuscript. BC, JZ, NW, SW, ZY, and FX performed the experiments.

## Supporting information

Supplementary Material

## Data Availability

The data that support the findings of this study are available from the corresponding author upon reasonable request.
