# Supplementary material for: Neuronal loss and microgliosis are restricted to the core of Aβ deposits in mouse models of Alzheimer's disease
Source: Aging Cell. 2021 May 25;20(6):e13380. doi: 10.1111/acel.13380 (PMC8208784; doi:10.1111/acel.13380)
Supplement: Supplementary file 1 — Supplementary Material [file ACEL-20-e13380-s001.docx]

Supplementary Figure

**
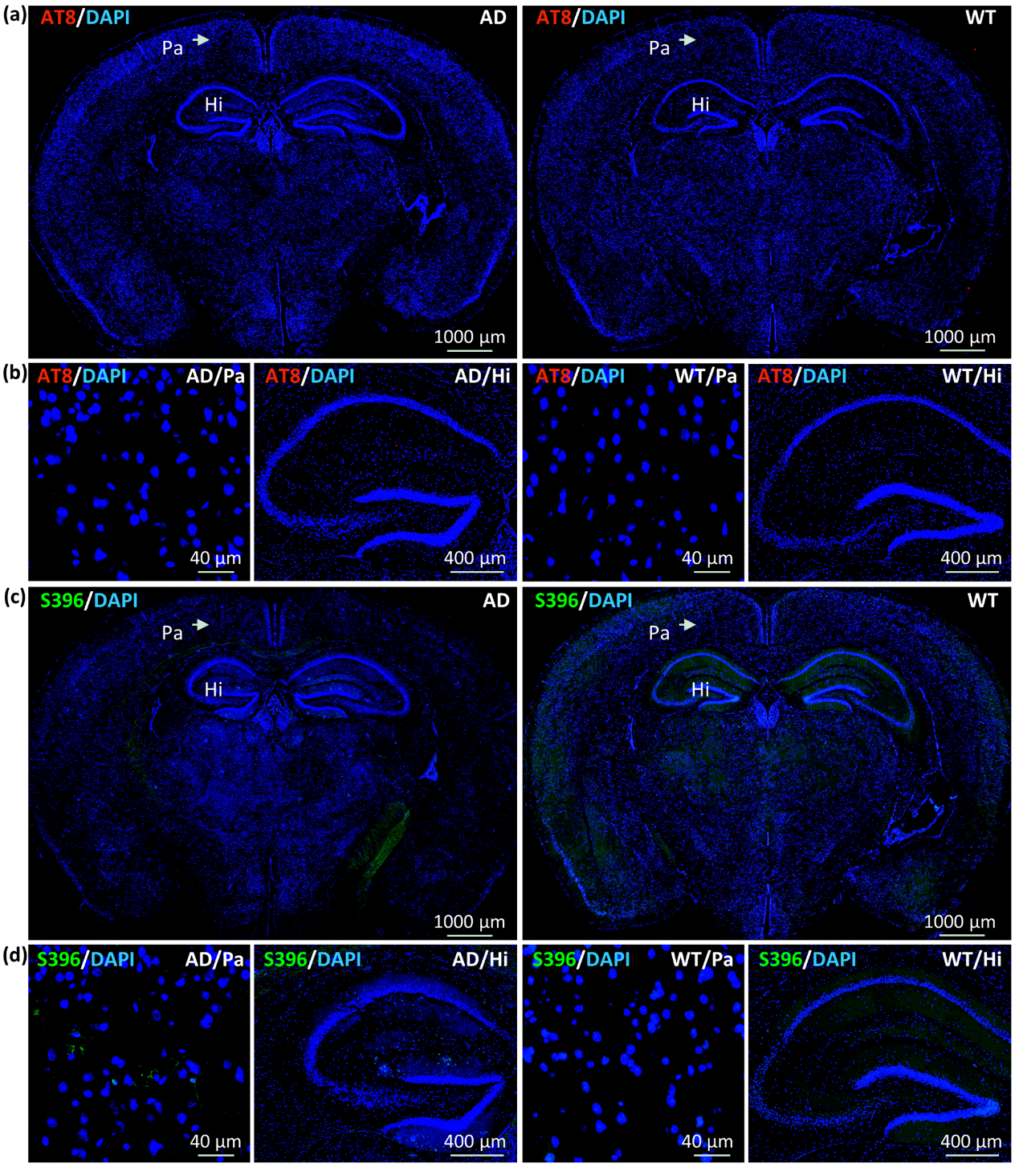
**

**Supplementary Figure 1. Pathologic tau was not detected in brain tissue from 12-**

**month-old 5xFAD mice**

(a) Representative images of immunofluorescence staining for AT8 (red), a phospho-tau (S202, T205) antibody, with DAPI counterstaining (blue) in coronal brain sections from 12-month-old 5xFAD and WT mice. Scale bars, 1000 μm. Areas in the parietal cortex (Pa) and hippocampus (Hi), indicated by arrows, are shown in (b) at high magnification. The scale bars for the Pa and Hi are 40 μm and 400 μm, respectively. No clear AT8 staining for pathologic tau was detected. (c) Brain sections from 5xFAD and WT mice were stained with a phospho-tau (S396) antibody (green) and counterstained with DAPI (blue). Scale bars, 1000 μm. Areas in the Pa and Hi, indicated by arrows, are shown in (d) at high magnification. The scale bars for the Pa and Hi are 40 μm and 400 μm, respectively. No clear S396 staining for pathologic tau was detected.

**
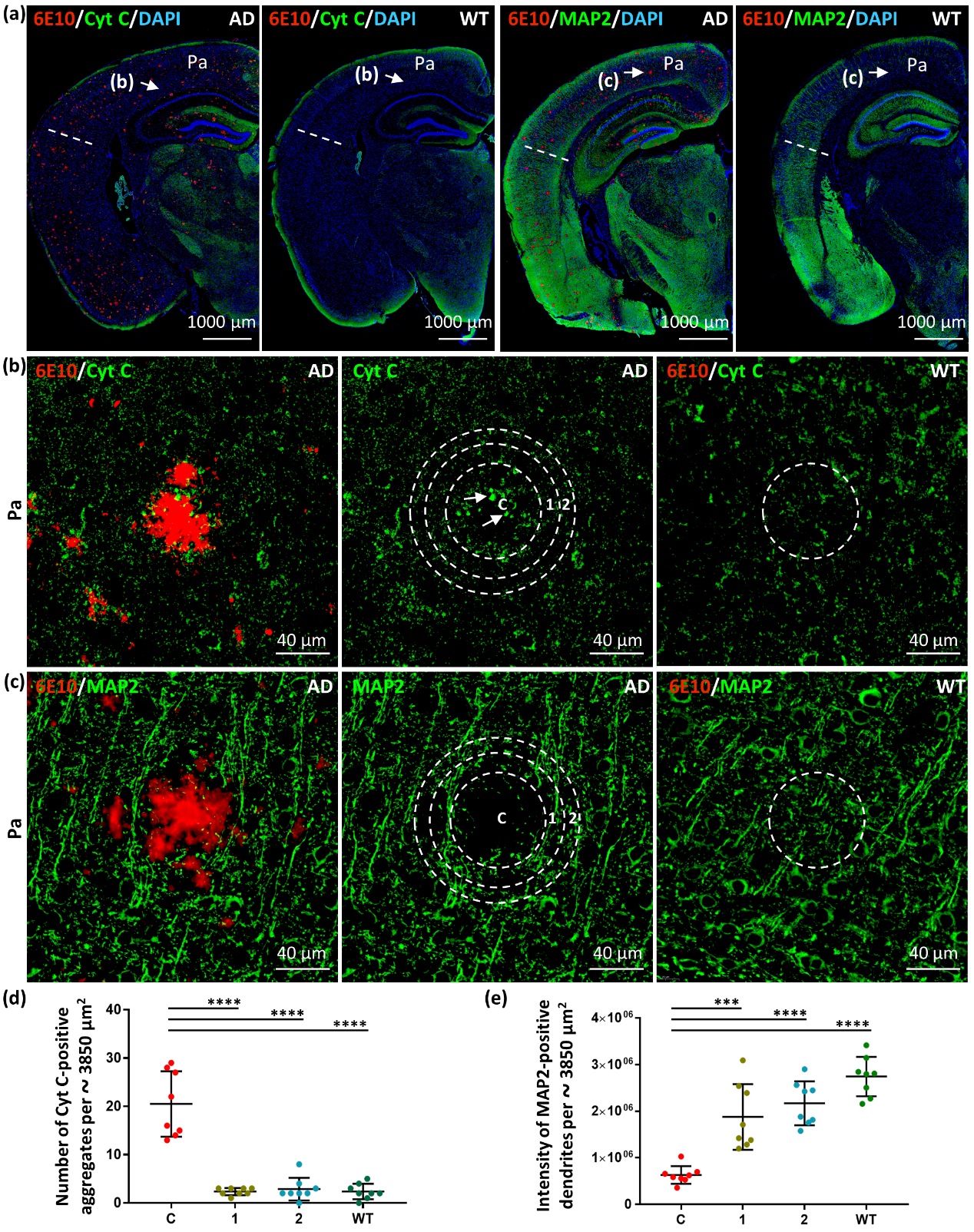
**

**Supplementary Figure 2. CytC-positive aggregate formation and MAP2-positive dendrite loss are restricted to the core area of Aβ deposits**

(a) Representative double-label immunofluorescence staining of 6E10 (red, a marker for Aβ deposits)/Cyt C (green, a marker for cellular death) or /MAP2 (green, a marker for dendrites) from 12-month-old 5xFAD and WT mice. Nuclei were counterstained with DAPI (blue). Scale bars, 1000 µm. Representative high magnification images in the parietal cortex (Pa) are shown in b and c, respectively. Scale bars, 40 µm. (b) Cyt C-positive aggregates mainly formed in the core of Aβ deposits compared with the two adjacent concentric rings in 5xFAD and WT mice. (c) In comparison with WT mice and the two adjacent concentric rings in 5xFAD mice, MAP2-positive dendrites in the core area of the Aβ deposits were fewer in number or even absent. (d) Quantitative analysis of the number of CytC-positive aggregates (per approximately 3850µm^2^) in the Pa in 5xFAD and WT mice. A significantly higher number of CytC-positive aggregates was found in C in comparison with 1, 2 or WT. (e) Quantitative analysis of the intensity of MAP2-positive dendrites (per approximately 3850µm^2^) in the Pa in 5xFAD and WT mice. MAP2 staining in C was significantly less intense in comparison with 1, 2 or WT. Data were derived from eight images of four different mice for each group (n = 8). Data represent mean ± SD. ****p* < 0.001; *****p* < 0.0001.


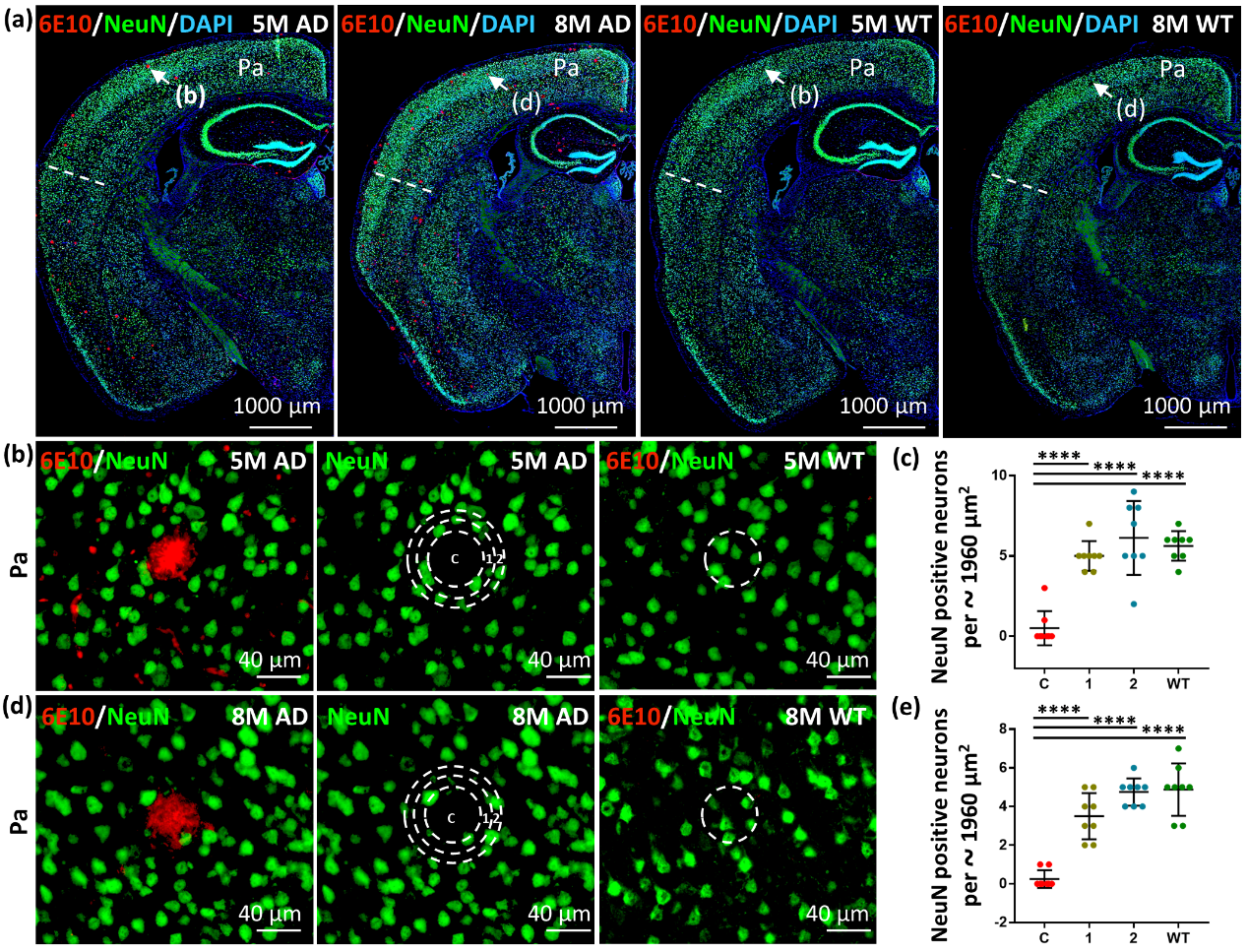


**Supplementary Figure 3.** **NeuN-positive neuronal loss is restricted to the core area of Aβ deposits in 5xFAD mice at young ages**

(a) Representative images of 6E10 (red)/NeuN (green) double-label immunofluorescence staining from 5-month-old (5M) and 8-month-old (8M) 5xFAD and age-matched WT mice. Nuclei were counterstained with DAPI (blue). Scale bars, 1000 µm. Representative high magnification images of NeuN-positive neurons and Aβ deposits in the parietal cortex (Pa) from 5M 5xFAD and WT mice (b), as well as from 8M 5xFAD and WT mice (d). Compared with age-matched WT mice and two adjacent concentric rings (1, 2), NeuN-positive neuronal loss was restricted to the core area of Aβ deposits in 5xFAD mice at the ages of 5 months and 8 months. The number of NeuN-positive neurons present in the core circle significantly decreased, (c and e, respectively). Scale bars, 40 µm. Data were derived from eight images of three different mice for each group (n = 8). Data represent mean ± SD. *****p* < 0.0001.

**
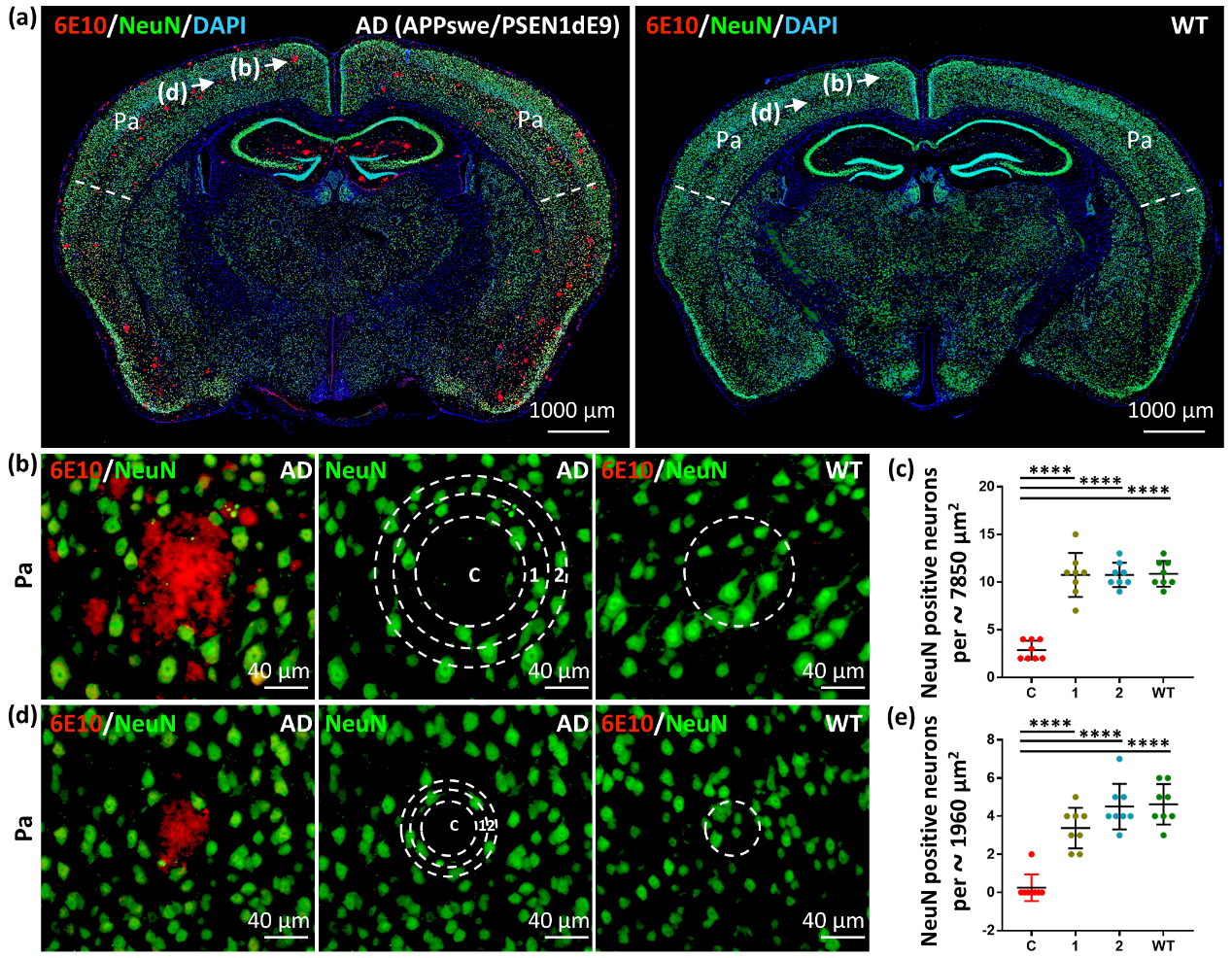
**

**Supplementary Figure 4.** **NeuN-positive neuronal loss is restricted to the core area of Aβ deposits in aged APPswe/PSEN1dE9 mice**

(a) Representative images of 6E10 (red)/NeuN (green) double-label immunofluorescence staining from 12-month-old APPswe/PSEN1dE9 and WT mice. Nuclei were counterstained with DAPI and are shown in blue. Scale bars, 1000 µm. Representative high magnification images of NeuN-positive neurons and large Aβ deposits (approximately 100 µm in diameter) in (b) or small Aβ deposits (approximately 50 µm in diameter) in (d) in the parietal cortex (Pa) from 12-month-old APPswe/PSEN1dE9 and WT mice. In comparison with age-matched WT mice and two adjacent concentric rings (1, 2), NeuN-positive neuronal loss was restricted to the core area of Aβ deposits in APPswe/PSEN1dE9 mice. The number of NeuN-positive neurons present in the core circle significantly decreased, as shown in (c) and (e). Scale bars, 40 µm. Data were derived from eight images of three different mice for each group (n = 8). Data represent mean ± SD. *****p* < 0.0001.
